# Comparison of clinical efficacy of different exercise therapies in the treatment of chronic nonspecific neck pain: a network meta-analysis

**DOI:** 10.3389/fneur.2026.1781903

**Published:** 2026-04-13

**Authors:** Min Huang, Haitao Liu, Jing Chen, Ren Pan

**Affiliations:** 1 College of Medical Imaging Laboratory and Rehabilitation, Xiangnan University, Chenzhou, Hunan, China; 2 The Affiliated Dongguan Songshan Lake Central Hospital, Guangdong Medical University, Dongguan, Guangdong, China

**Keywords:** chronic non-specific neck pain, exercise therapy, muscle energy technique, neck dysfunction index, network meta-analysis, visual analogue scale

## Abstract

**Background:**

Chronic nonspecific neck pain (CNSNP) is a common musculoskeletal disorder that significantly impacts patients’ quality of life and imposes a considerable social and economic burden. Although drug treatment is widely used, it has limitations such as adverse reactions and limited long-term efficacy. Therefore, exercise therapy has gradually become the first-line intervention method. However, there is still a lack of systematic evidence for the efficacy comparison among different exercise therapies.

**Objective:**

This study conducted a network meta-analysis to compare the clinical efficacy of different exercise therapies in treating CNSNP, providing evidence support for clinical intervention selection.

**Methods:**

Systematic searches were conducted in PubMed, Web of Science, Embase, Cochrane Library, VIP, SinoMed, CNKI, and Wanfang databases up to November 25, 2025, for randomized controlled trials comparing the efficacy of different exercise therapies in patients with CNSNP. Inclusion criteria were studies comparing the efficacy of different exercise therapies in patients with CNSNP. The primary outcome measures were the Neck Disability Index (NDI) and Visual Analogue Scale (VAS). The quality of studies was evaluated using the Cochrane Risk of Bias Assessment Tool 2.0. Network meta-analysis was performed using Stata 16.0 and R 4.5.2, and the intervention measures were ranked using the cumulative ranking area under the curve (SUCRA). The evidence quality was evaluated using the CINeMA tool.

**Results:**

A total of 17 studies were included, involving 1,224 patients and 16 intervention measures. The risk of bias assessment showed that 12 studies (70.6%) were of moderate risk, and 5 studies (29.4%) were of high risk. There was no significant difference in improving neck function (NDI) among the intervention measures. The SUCRA value of BBAT (biological feedback-assisted training) was the highest (0.78), but this result was supported by only a small sample of single studies, and the robustness was limited. In terms of reducing pain (VAS), muscle energy technique (MET) was significantly superior to conventional exercise therapy (*p* < 0.05), with a SUCRA value of 0.88. However, sensitivity analysis showed that this result was somewhat dependent on a single study. Meta-regression showed that the treatment duration had no significant effect on the efficacy. Publication bias analysis did not reveal significant bias. The CINeMA evidence quality evaluation showed that the overall credibility of the current evidence was moderate.

**Conclusion:**

Muscle energy technique (MET) shows certain advantages in alleviating chronic nonspecific neck pain, but there is no significant difference among the intervention measures in improving neck function. Due to the sparse network connection of some intervention measures and the reliance on indirect evidence in most comparisons, the SUCRA ranking results should be regarded as exploratory findings and are not sufficient to form clear clinical recommendations. Future high-quality, large-sample, and long-term follow-up head-to-head randomized controlled trials are needed to further verify the efficacy differences of different exercise therapies.

**Systematic review registration:**

https://www.crd.york.ac.uk/PROSPERO/view/CRD420251249215.

## 1 Introduction

Chronic non-specific neck pain (CNSNP), as a common musculoskeletal disorder, presents a significant epidemiological burden worldwide. With the changes in modern lifestyle, especially the increase in prolonged use of electronic devices and sedentary behavior, the prevalence of CNSNP continues to climb. Studies have shown that the incidence of CNSNP is particularly prominent in the young population aged 18–29 years Jahre et al. (1). This pain not only affects patients’ work efficiency and quality of daily life, but also causes a huge socio-economic burden, including direct medical expenses and indirect productivity loss (2). It is worth noting that CNSNP tends to show a chronic trend, and about 30% of patients will develop into a chronic pain state, further increasing the burden on the medical system (3).

The mechanism of cervical spine pain is complex and involves various pathological and physiological changes in multiple structures. In recent years, the role of neuropeptides in the occurrence and maintenance of cervical pain has gradually attracted attention. Studies have shown that in cervical nerve root injury models, the expression of substance P and calcitonin gene-related peptide (CGRP) in the spinal dorsal horn significantly increases, and their dynamic changes are parallel to the behavioral manifestations of mechanical hyperalgesia, suggesting that these neuropeptides may play a key role in the pathogenesis of cervical pain. In addition, there are abundant CGRP and substance P positive sensory nerve fibers in the cervical dura mater and posterior longitudinal ligament, and the activation of these neurons may participate in the conduction and maintenance of cervical pain. Besides endogenous neuropeptides, exogenous peptides as potential therapeutic strategies are also being explored. For example, BPC-157 is a natural polypeptide derived from human gastric juice, and preclinical studies have shown that it can promote tendon, ligament, and other soft tissue repair and reduce inflammatory responses. In a retrospective study, 7 out of 12 patients with chronic knee pain experienced pain relief for more than 6 months after intra-articular injection of BPC-157. Moreover, synthetic peptides such as SP16, derived from α1-antitrypsin, have shown inhibitory effects on inflammatory pain and neuropathic pain in animal models. Although current research on peptide-based treatments for cervical pain is still in its early stages, these findings provide potential directions for the development of new, non-opioid pain intervention strategies (4).

As an important means of treating CNSNP tumors, the advantages of drug therapy are mainly reflected in the rapid relief of symptoms and improvement of function. Non-steroidal anti-inflammatory drugs (NSAIDs) and skeletal muscle relaxants (SMRs) are widely used for pain control in the acute phase. The systematic review by Oldfield et al. showed that tizanidine and other SMRs had significant effects on patients with neck pain and muscle spasms (5). Adjusted doses of NSAIDs combined with gastrointestinal protection can provide relatively safe pain relief in elderly patients (6). However, drug therapy has obvious limitations: even short-term use of opioids has no significant difference in the efficacy of acute neck pain compared with placebo, but it increases the risk of adverse reactions such as constipation (7). The problem of drug metabolism is particularly prominent in long-term use. Elderly patients are more prone to cumulative toxicity due to reduced liver and kidney function, and the interaction caused by multiple drugs may counteract the therapeutic effect (8). In addition, Zaina’s study on lumbar spinal stenosis suggested that the effect of conservative treatment (including drugs) was equivalent to that of surgery within 6 months, but the long-term effect may be worse than invasive intervention (9). This finding questioned the long-term value of drug management of neck pain, and more and more doctors turned their attention to non-drug therapies.

As an important intervention for CNSNP, exercise therapy has been widely recognized in clinical practice in recent years. The systematic review of European clinical practice guidelines showed that exercise therapy and manual therapy together constitute the core non-pharmacological intervention program for neck pain management, and its recommendation strength has reached a moderate level (10). Compared with drug therapy, the advantage of exercise therapy is that it can continuously improve neck function, reduce the degree of disability, and has no drug-related adverse reactions. Systematic reviews have confirmed that multimodal interventions that combine exercise therapy with manual therapy are significantly more effective than single exercise intervention (11). However, there are still some controversies and limitations in the clinical application of exercise therapy. First of all, there is a lack of high-quality evidence for the comparison of the effects of different exercise programs, and the existing studies generally have methodological defects such as insufficient control group Settings (12). Secondly, there is no consensus on the optimal dose, intensity and specific implementation plan of exercise intervention, and the comparison of the advantages and disadvantages between different techniques such as postural re-education and Pilates is still inconclusive (13). In addition, factors such as poor patient compliance, high treatment cost and the need for professional guidance also limit the wide application of exercise therapy to a certain extent. It is worth noting that the assessment of cervical spine range of motion, muscle strength, sensory function, and deep cervical flexor endurance is of great significance for determining the degree of functional impairment and formulating individualized exercise prescriptions (14). In the clinical application of exercise therapy, the identification of contraindications is of crucial importance. For patients with severe osteoporosis, uncontrolled cervical instability, acute intervertebral disc protrusion with progressive neurological deficits, recent history of cervical surgery, severe cardiovascular diseases or malignant tumors, the initiation of exercise therapy requires careful assessment. In some cases, it should be postponed or avoided (15). Moreover, for patients with obvious psychological disorders or severe fear-avoidance behaviors towards exercise, simple exercise intervention may not be effective. Combined cognitive-behavioral intervention is needed to improve compliance and efficacy (16). Therefore, in the selection and implementation of exercise therapy, fully assessing the indications and contraindications of the patients is the prerequisite for ensuring safety and effectiveness.

Although exercise therapy has been established as a first-line intervention for CNSNP, there is significant heterogeneity and methodological limitations in the available evidence. Traditional pairwise meta-analysis can only compare the relative efficacy of two interventions, while in clinical practice, it is often necessary to choose between various exercise regimens, such as yoga, Pilates, stability training, resistance training, and other exercise therapies. By integrating direct and indirect comparative evidence, network meta-analysis can construct a hierarchical ranking of the effectiveness of multiple interventions and provide a more comprehensive basis for clinical decision-making.

## 2 Methods

### 2.1 Register

This study has been prospectively registered with PROSPEO under the number CRD420251249215.

### 2.2 Inclusion criteria

(1) Population: patients with chronic non-specific neck pain. Age, gender, course of disease and race were not limited. (2) Intervention: exercise therapy (including strength training, aerobic exercise, stretching training, muscle energy techniques, stability training/motion control, virtual reality training, comprehensive exercise training, etc.) or combined with acupuncture, manipulation, health education, etc. (3) control group: exercise therapy or placebo. (4) Outcome measures: Neck Disability Index (NDI) (17): The neck Disability Index (NDI) is the most widely used instrument for measuring the degree of self-reported disability due to neck pain (18). It is a self-report instrument with 10 items: pain severity, personal care, lifting, work, headache, concentration, sleep, driving, reading, and recreation. Each answer is rated on a 6-point scale ranging from 0(no disability) to 5(severe disability). A higher score indicates a greater degree of disability. The Visual Analog Scales (VAS) score (19): is a commonly used subjective tool to assess the intensity of pain. Scores range from 0 to 10, with lower scores indicating less pain. Reporting of an outcome measure was eligible for inclusion. (5) Study type: randomized controlled trial. The language was limited to Chinese and English.

### 2.3 Exclusion criteria

(1) Duplicate literature. (2) Non-randomized controlled trials (reviews, basic trials, abstracts, etc.). (3) Inconsistent interventions. (4) Incomplete or erroneous data. (5) Literature could not be obtained. (6) Could not form a network meta-analysis.

### 2.4 Search strategy

A systematic search was conducted in PubMed, Web of Science, Embase, Cochrane Library, VIP, SinoMed, CNKI, and Wanfang databases for randomized controlled trials investigating exercise therapy for chronic non-specific neck pain. A combination of MeSH terms and free-text words was used. The search period covered from database inception to November 25, 2025 (Supplementary Material S1: Search Strategy).

### 2.5 Literature screening and data extraction

Two researchers independently screened the literature using Endnote. After duplicate checking, reading title and abstract for preliminary screening, and then reading the full text for re-screening, the included studies were determined. Excel was used to enter the data. The extracted contents included ① basic information: author, journal, region, and publication year. ② Baseline data: patient information, interventions, treatment course, and outcome indicators. ③ Risk of bias information: randomized method, allocation concealment, blinding, etc. The authors of the original study were contacted for detailed data if the data were incomplete. Check with each other after completion, and if there are any questions, discuss the decision within the group.

### 2.6 Risk of bias assessment

The risk of bias was assessed by two reviewers independently using the Cochrane Risk of bias assessment tool ROB2 (20). Five domains were assessed: bias in the randomization process, bias of deviation from the intended intervention, bias of missing outcome data, bias of outcome measurement, and bias of selective reporting of outcomes. The risk of bias in each domain can be divided into three levels: low risk, unknown risk and high risk. If the results of the risk of bias assessment in all domains are “low risk,” then the overall risk of bias is “low risk”; if the results of the risk of bias assessment in some fields were “moderate risk” and there were no “high risk” areas, then the overall risk of bias was “moderate risk.” As long as there is only one domain with “high risk,” the overall risk of bias is “high risk.” Two investigators independently evaluated and compared the results. In case of disagreement, a third researcher was consulted or the decision was discussed.

### 2.7 Statistical analysis

Stata16.0 and R4.5.2 were used for meta-analysis. Relative risk (RR) was used for binary variables, and standardized mean difference (MD) was used for continuous variables, and the credibility interval (CI) was calculated. The I^2^ statistic test was used to determine the degree of heterogeneity. When I^2^ ≤ 50%, there was no statistical heterogeneity. When I^2^ > 50%, it indicated statistical heterogeneity, and sensitivity analysis was performed to explore the source of heterogeneity. The evidence network diagram of each outcome index was drawn. When a closed loop appeared, the inconsistency test was performed. If *p* > 0.05, the consistency model was used for analysis. If *p* < 0.05, the inconsistency was reported, and the inconsistency was tested by the node-splitting method. Each outcome measure was ranked to obtain the area under the cumulative ran-king (SUCRA) curve. SUCRA was expressed as a percentage, with higher percentages indicating better interventions. When the number of articles included in the outcome index was more than 10, the comparison-correction funnel plot was drawn and publication bias test was conducted to determine whether there was publication bias and small sample effect. Meta-regression was used to explore the factors affecting the results.

### 2.8 Evaluation of evidence quality

The evidence quality was evaluated using CINeMA (Confidence in Network Meta-Analysis). CINeMA is a tool for evaluating the quality of evidence in network meta-analysis, developed based on the GRADE framework. It enables semi-automated evaluation through an online application (https://cinema.ispm.unibe.ch) (21). This tool assesses the results of network meta-analysis in six domains: internal bias of the study, reporting bias, indirectness, imprecision, heterogeneity, and inconsistency. It quantifies the weight of each original study on the combined effect size using a contribution matrix, and finally, it synthesizes the results to obtain four levels of evidence quality: “high,” “medium,” “low,” or “very low.” Compared to the traditional GRADE method, CINeMA is particularly suitable for network meta-analyses with numerous intervention measures and complex network structures. In this study, this tool will be used to grade the evidence quality of the primary outcome indicators (NDI and VAS).

## 3 Results

### 3.1 Study selection

**Figure 1:**
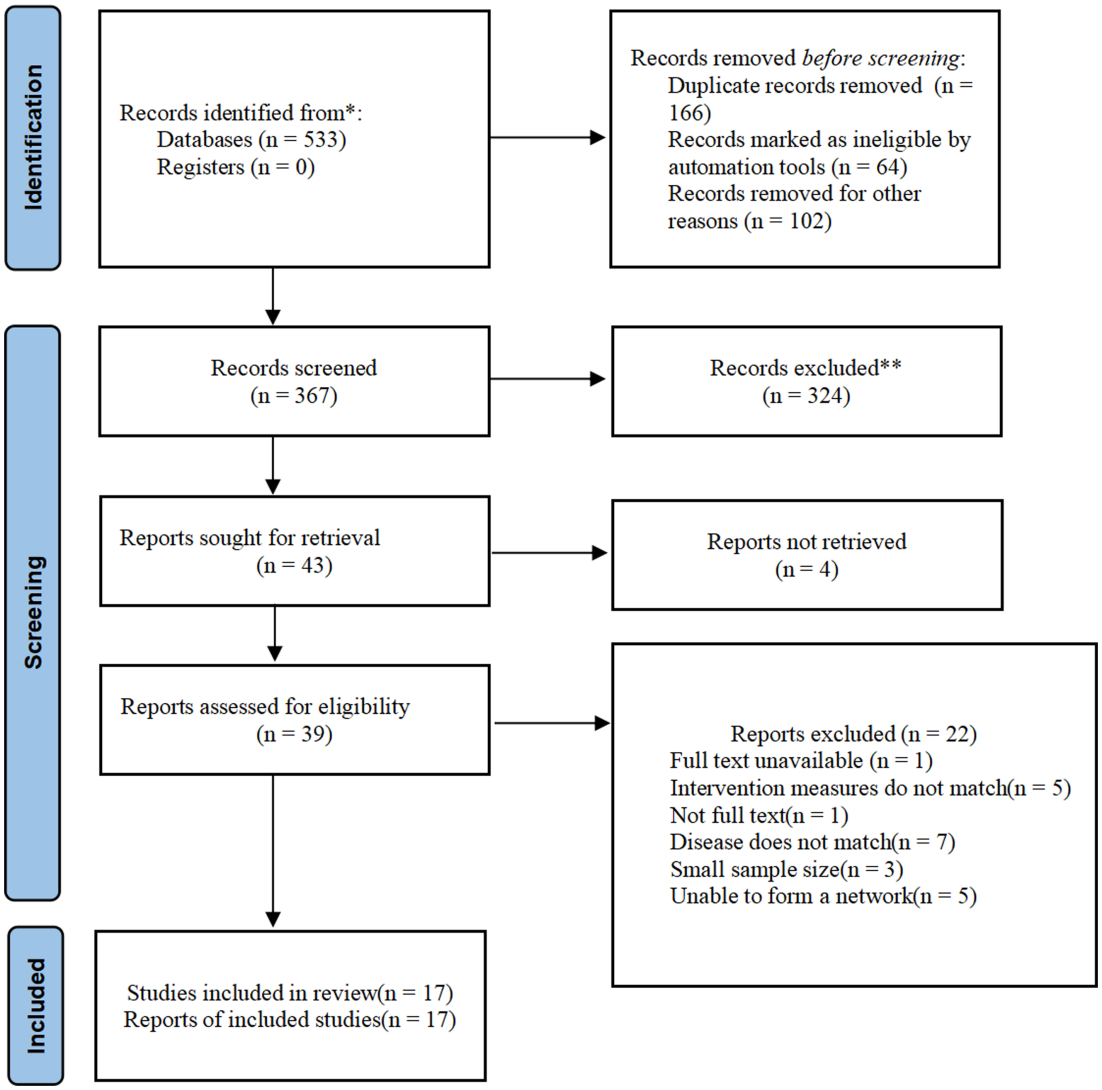
PRISMA flow chart (*Consider, if feasible to do so, reporting the number of records identified from each database or register searched rather than the total number across all databases/registers.**if automation tools were used, indicate how many records were excluded by a human and how many were excluded by automation tools).

A total of 533 articles were retrieved, and 17 studies were finally included after screening. Figure 1 is the screening flow chart.

### 3.2 Study characteristics

Seventeen studies (18, 22–37) with a total of 1,224 patients were included in this study. There were 16 interventions: isometric exercise (IE), tailored therapyt (TT), Non-tailored therapy (NTT), Muscle energy technique (MET), Myofascial release therapy (MRT), Conventional exercise (CE) + Traditional Chinese medicine (TCM), Suspension exercise (SE) + CE, Basic Body Awareness Therapy (BBAT), Osteopathic manipulative therapy (OMT) + CE, Back school (BS), Dynamic taping (DT), Extracorporeal shock wave therapy (ESWT) + CE, Jaw opening (JO) + CE, Active behavioural physiotherapy (ABP,) Specifc cervical extensors exercise (SCEE), CE. Table 1 presents the basic characteristics of the included studies.

**Table 1:** The basic characteristics included in the study.

Study author	Country	Year	Intervening measure	Sample size	Study design	Time	Outcome indicator
Muhammad Khan (22)	Pakistan	2014	IE/CE	34/34	Randomised controlled trail	12 weeks	VAS
Åsa Svedmark (23)	Sweden	2016	TT/NTT/CE	40/40/40	Single-center, single-assessor blinded ran-domized controlled clinical trial	12 weeks	NDI
Carlos Bernal-Utrera (24)	Spain	2020	TE/Placebo	23/23	Randomized, controlled, parallel, double-blind, three-arm clinical trial	12 weeks	NDI
Reema Joshi (25)	India	2022	MET/CE	23/25	Randomized, single blinded, clinical trial	2 weeks	NDI
Zainab Khalid Khan (26)	Pakistan	2022	MET/MRT	30/30	Single-blinded randomization	2 weeks	VAS, NDI
Yucui Chen (27)	China	2022	CE + TCM/CE	58/58	Randomized controlled clinical trial	4 weeks	VAS, NDI
Shasha Huang (28)	China	2022	SE + CE/CE	20/20	Randomized controlled clinical trial	8 weeks	VAS, NDI
Yousef M. Alshehre (29)	Saudi Arabia	2023	BBAT/CE	29/29	Single-blinded, randomized, controlled trial	8 weeks	NDI
Sandro Groisman (30)	Brazil	2023	OMT + CE/CE	45/45	A randomized, controlled, dual-blind clinical trial	12 weeks	NDI
Pablo Hernandez-Lucas (31)	Spain	2023	BS/Placebo	29/29	A randomized controlled clinical trial	8 weeks	NDI
Mohammad Sidiq (18)	India	2023	DT/Placebo	69/67	A prospective parallel-group active controlled trial	4 weeks	VAS, NDI
Hongkun Wang (32)	China	2023	ESWT+CE/CE	30/30	Randomized controlled clinical trial	3 weeks	VAS
Saeed Akhter (33)	Pakistan	2024	JO + CE/CE	40/40	Randomized controlled trial with a double-blinded, two-armed, parallel design	6 weeks	NDI
Taweewat Wiangkham (34)	Thailand	2024	ABP/CE	30/30	A pilot and feasibility trial of a pragmatic cluster randomised double-blind (assessors and participants)	4 weeks	NDI
Yao Zhang (35)	China	2024	SCEE/CE	35/35	Single-blind, single-center, prospective, randomized controlled trial	48 weeks	VAS, NDI
Lang Zhao (36)	China	2024	ESWT+CE/CE	30/30	Randomized controlled clinical trial	2 weeks	VAS, NDI
Kun Zheng (37)	China	2024	MET/CE	27/27	Randomized controlled clinical trial	1 week	VAS, NDI

CE, Conventional exercise; IE, isometric exercise; TT, tailored therapy; NTT, Non-tailored therapy; TE, Therapeutic exercise; MET, Muscle energy technique; MRT, Myofascial release therapy; TCM, Traditional Chinese medicine; SE, Suspension exercise; BBAT, Basic Body Awareness Therapy; OMT, Osteopathic manipulative therapy; BS, Back school; DT, Dynamic taping; ESWT, Extracorporeal shock wave therapy; JO, Jaw opening; ABP, Active behavioural physiotherapy; SCEE, Specifc cervical extensors exercise.

### 3.3 Risk of bias instudies

**Figure 2:**
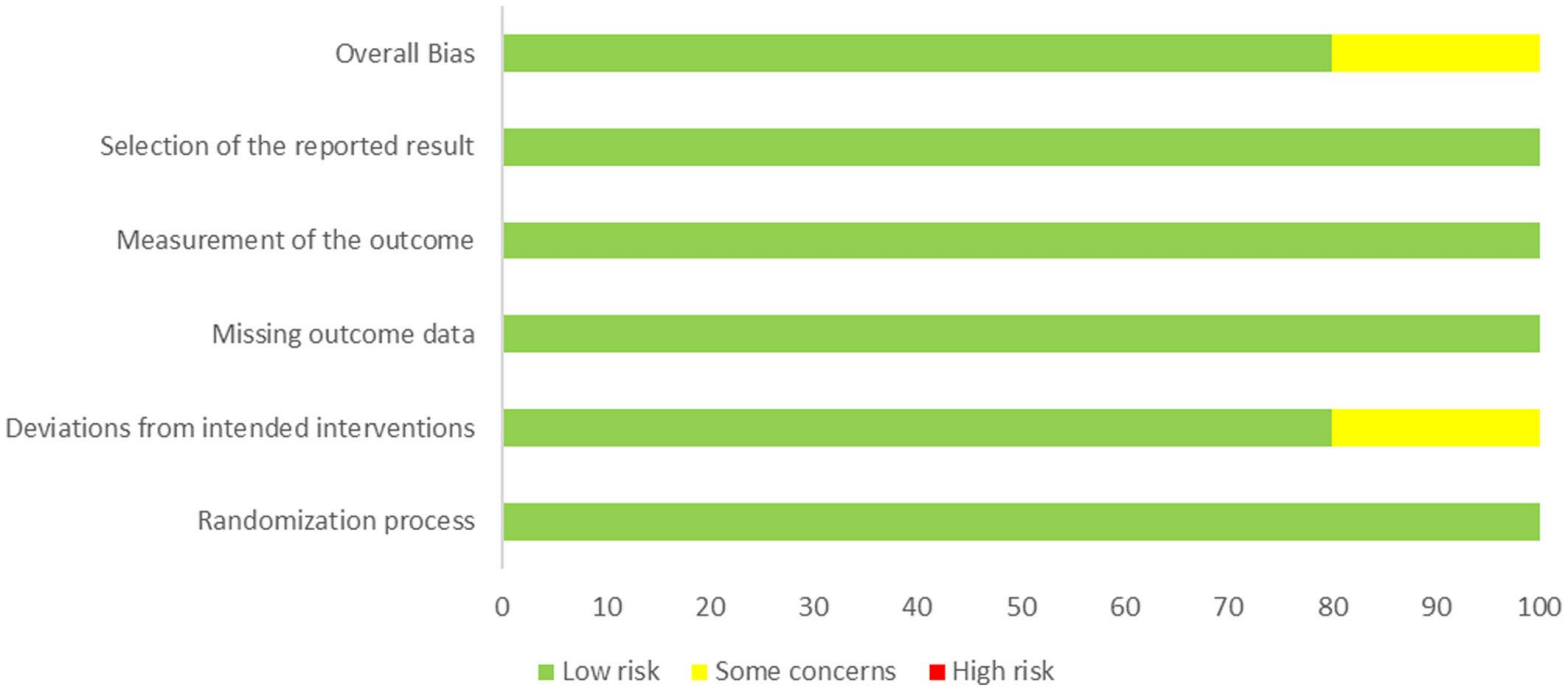
Results of risk of bias assessment for included studies.

Sixteen studies employed correct randomization methods, ten reported hidden allocation, and ten used blinding. All the study data were complete, and no selective reporting or other obvious sources of bias were found. The risk assessment of bias results showed that 12 studies (70.6%) were “moderate risk,” 5 studies (29.4%) were “high risk,” and no study was rated as “low risk.” Due to the particularity of the intervention measures in the randomized controlled trials related to exercise therapy, it is difficult to implement blinding for participants and therapists, which is the main reason for the “moderate risk” and “high risk” ratings (Figure 2).

### 3.4 Meta-analysis

#### 3.4.1 NDI

**Figure 3:**
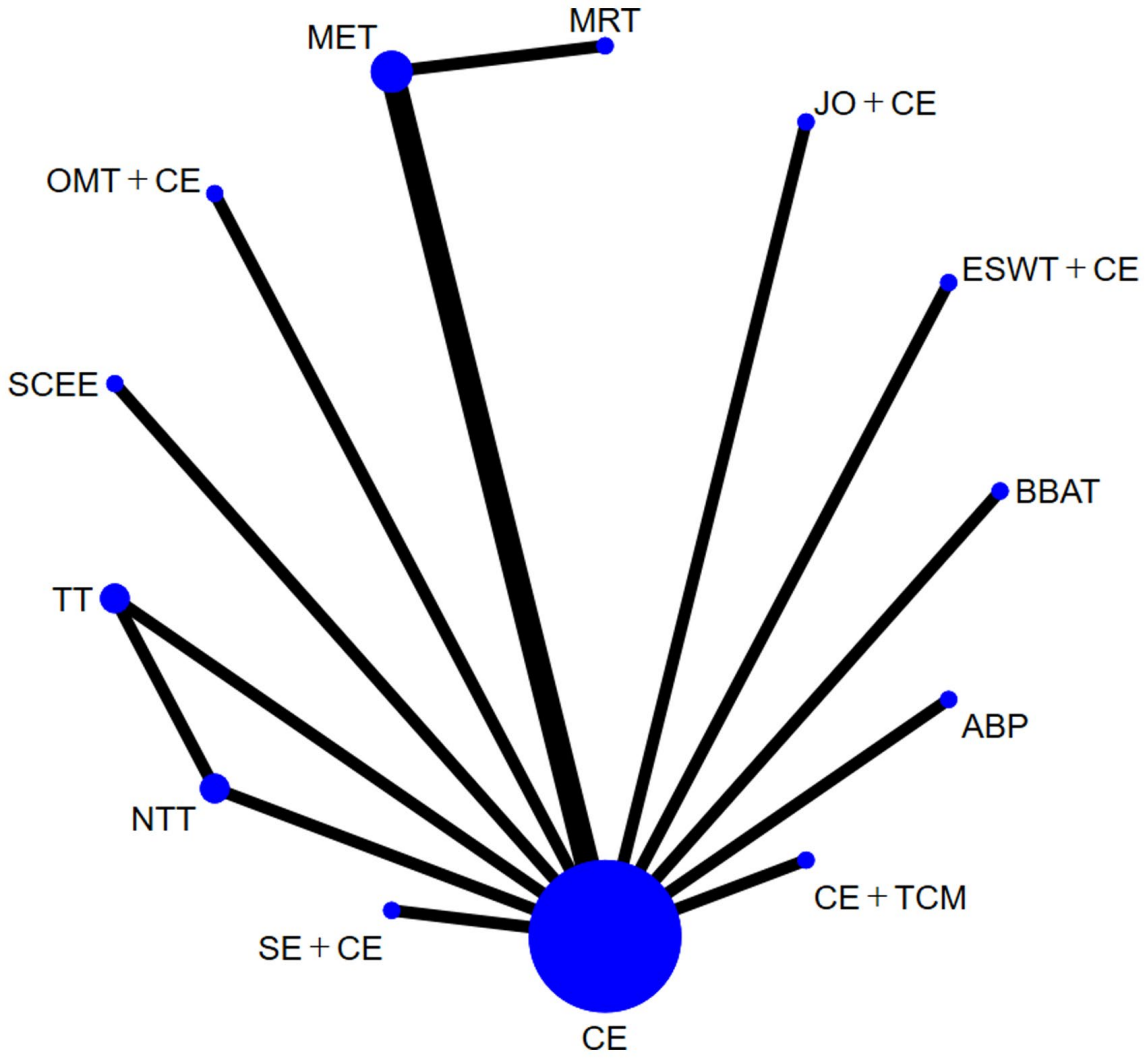
Evidence network diagram of interventions with neck disability index (NDI) as the outcome measure.

**Figure 4:**
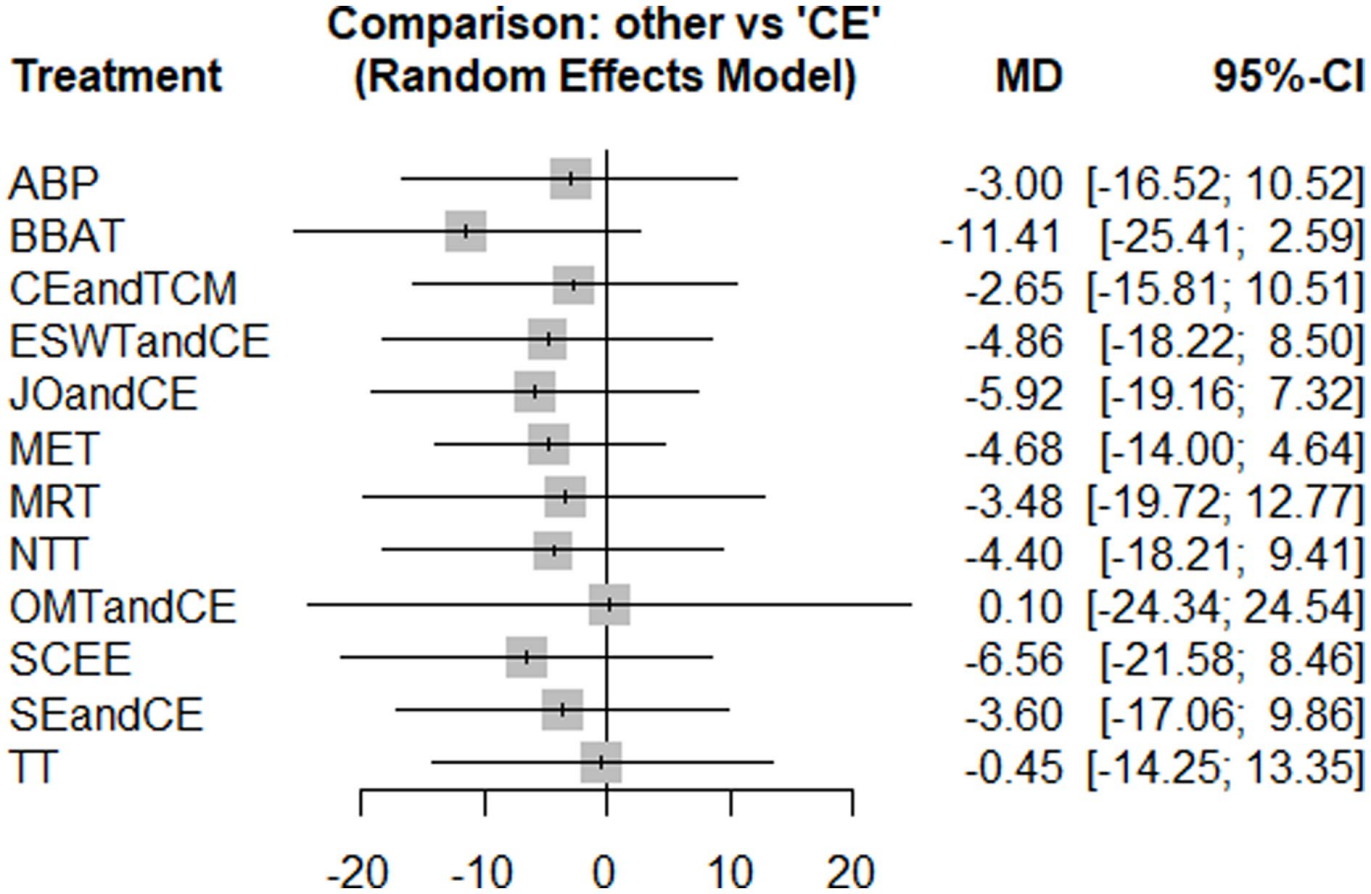
Results of the network meta-analysis of the effects of different interventions on NDI (forest plot).

NDI was reported in 15 studies with 12 treatments in the trial groups. There was a closed loop in the reticular diagram, and the inconsistency test results showed *p* > 0.05, which was analyzed using the consistency model (Figure 3). The results of heterogeneity test showed that I^2^ = 4%. The results of the network meta-analysis showed that there was no significant difference in effect between the interventions (Figure 4). Supplementary Material S2: League table). The top three ranked by SUCRA are: BBAT (0.78), SCEE (0.60), JOandCE (0.58) (Table 2). It is important to note that in the NDI aspect, BBAT has the highest SUCRA value (0.78), but this is only supported by a small sample study. Moreover, the analysis after excluding each item shows that the conclusions related to NDI are highly dependent on a single study and have limited robustness. Therefore, the ranking of BBAT should be interpreted with caution.

**Table 2:** SUCRA ranking table of different interventions for NDI and VAS outcomes.

Intervening measure	NDI		VAS	
	SUCRA	Rank	SUCRA	Rank
ABP	0.46	9		
BBAT	0.78	1		
CE	0.28	13	0.18	8
CEandTCM	0.44	10	0.58	3
ESWTandCE	0.54	5	0.30	7
JOandCE	0.58	3		
MET	0.54	4	0.88	1
MRT	0.48	8	0.67	2
NTT	0.52	6		
OMTandCE	0.38	11		
SCEE	0.60	2	0.34	6
SEandCE	0.48	7	0.52	4
TT	0.35	12		
IE			0.49	5

CE, Conventional exercise; IE, isometric exercise; TT, tailored therapy; NTT, Non-tailored therapy; TE, Therapeutic exercise; MET, Muscle energy technique; MRT, Myofascial release therapy; TCM, Traditional Chinese medicine; SE, Suspension exercise; BBAT, Basic Body Awareness Therapy; OMT, Osteopathic manipulative therapy; BS, Back school; DT, Dynamic taping; ESWT, Extracorporeal shock wave therapy; JO, Jaw opening; ABP, Active behavioural physiotherapy; SCEE, Specifc cervical extensors exercise.

#### 3.4.2 Vas

**Figure 5:**
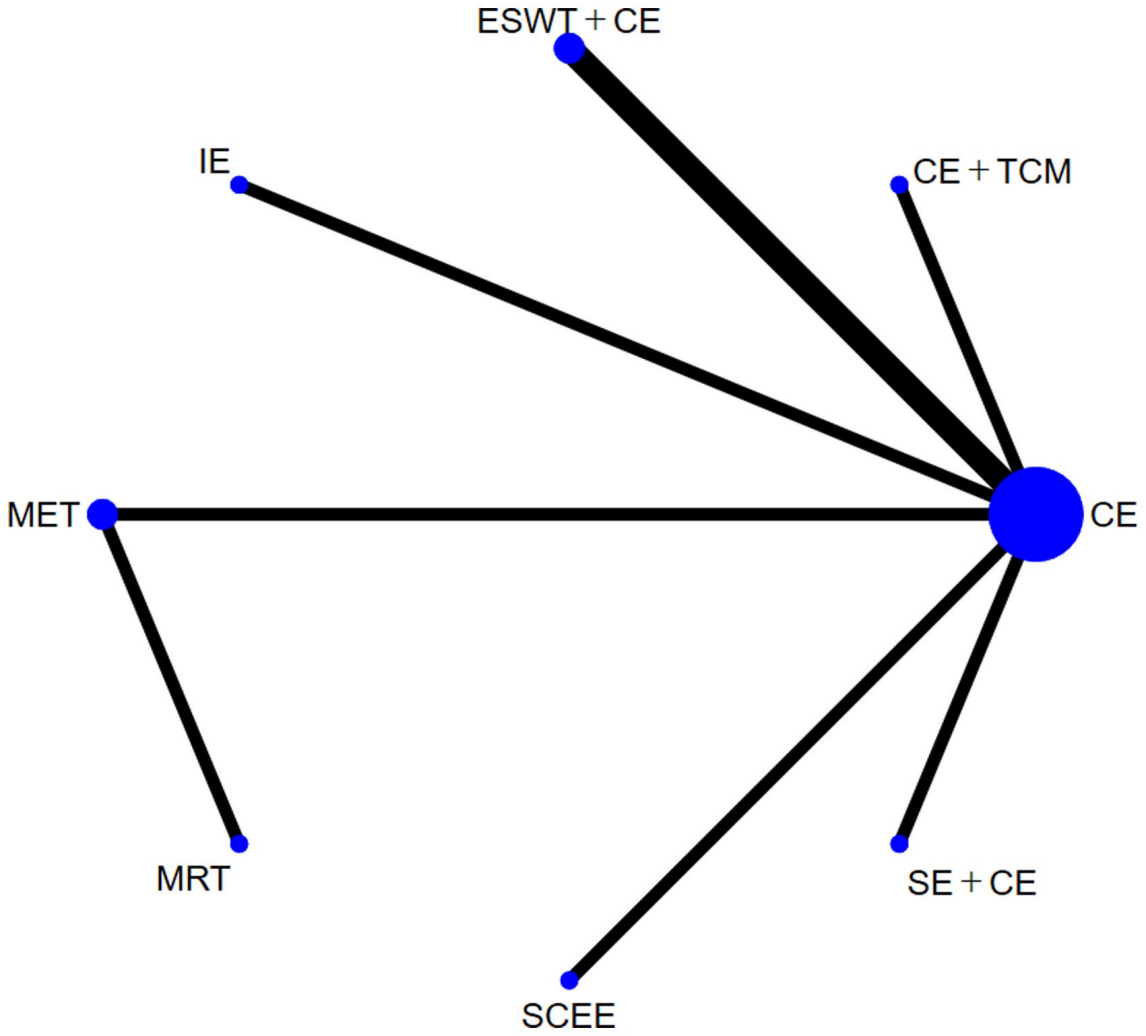
Evidence network diagram of interventions with visual analogue scale (VAS) as the outcome measure.

**Figure 6:**
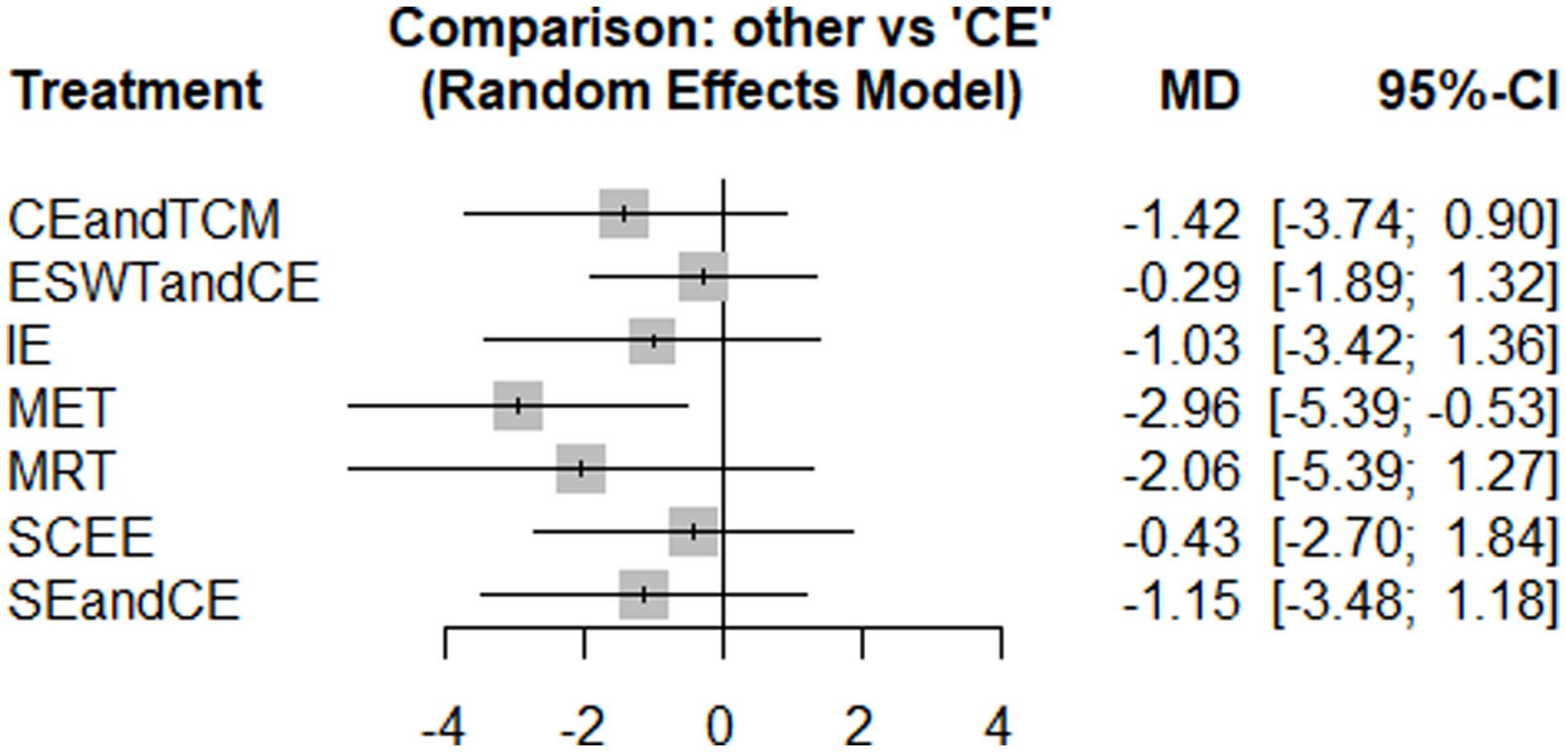
Results of the network meta-analysis of the effects of different interventions on VAS (forest plot).

Nine studies reported VAS, and the experimental groups included seven treatments. The reticular plots did not have closed loops and were analyzed using a consistency model (Figure 5). The results of the heterogeneity test showed I^2^ = 7%. The results of network meta-analysis showed that the effect of MET was significantly better than that of conventional exercise (*p* < 0.05) (Figure 6). There was no significant difference in effectiveness between the interventions (Supplementary Material S2: League table). The top three SUCRA rankings were MET (0.88), MRT (0.67), and CE and TCM (0.58) (Table 2). In the SUCRA ranking, some intervention measures (such as BBAT, SCEE, JO + CE, etc.) are supported by only one small-sample study. Although their rankings are numerically high, due to the weak evidence base, the ranking results are unstable and have limited clinical reference value. In contrast, the SUCRA value of MET in the VAS aspect (0.88) is based on multiple studies and the results of the sensitivity analysis are stable, indicating more sufficient evidence support.

#### 3.4.3 Analysis of publication bias

**Figure 7:**
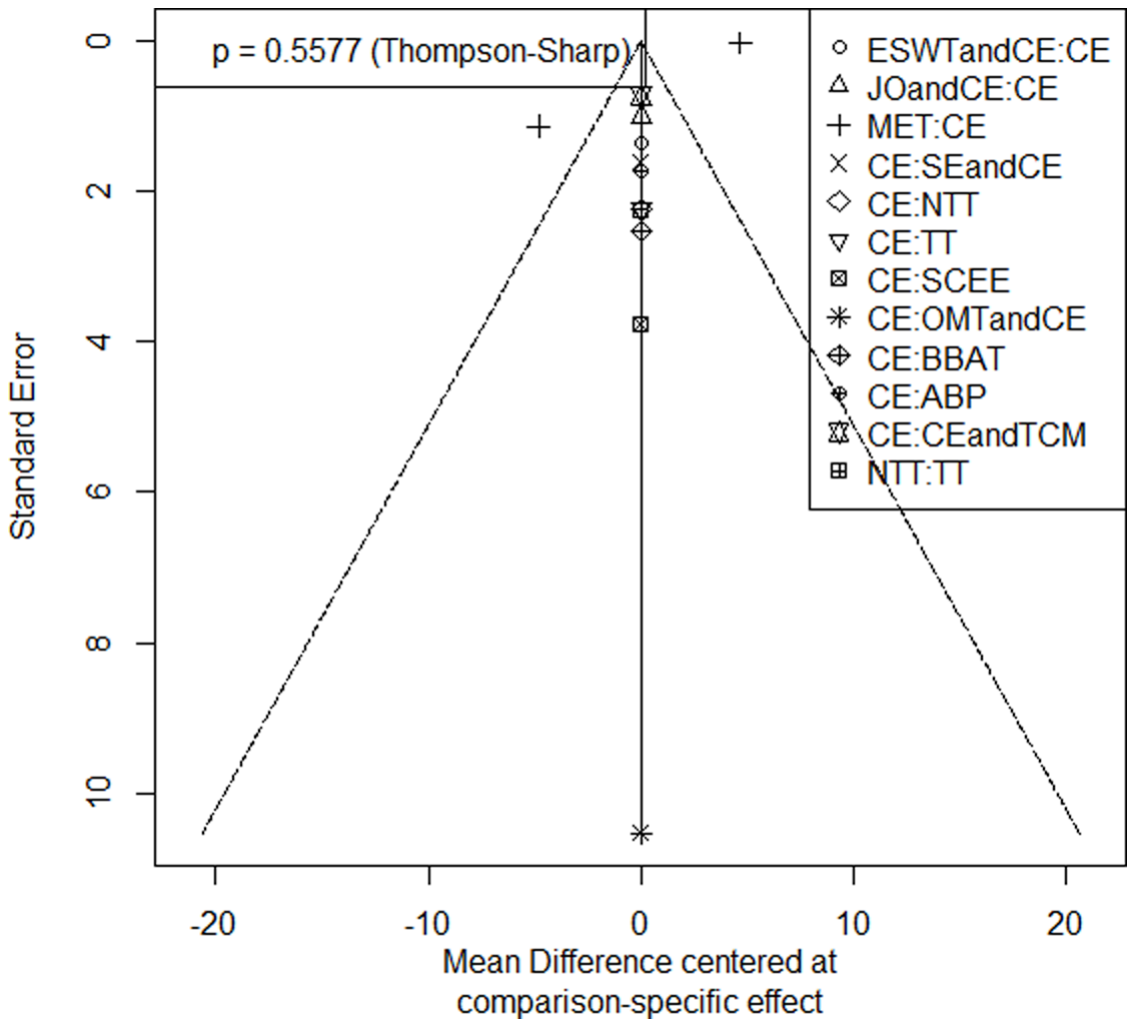
Funnel plot of publication bias for NDI outcome measures.

The results of NDI were tested for publication bias, and the results showed a small possibility of publication bias (*p* = 0.55) (Figure 7).

#### 3.4.4 Sensitivity analysis and Meta-regression

We conducted a sensitivity analysis: We excluded studies that explicitly included non-exercise therapy components such as acupuncture, traditional Chinese medicine, and massage, and only retained intervention measures centered on pure exercise therapy. The results showed that on the NDI outcome indicators, the results remained stable after eliminating each study one by one. On the VAS outcome indicators, after excluding Zainab Khalid Khan’s study, the comparison among the various intervention measures changed from no statistical significance to statistically significant. The characteristics of this study are: the effect size is significantly higher than that of other studies, and it provides direct comparison evidence of MET and MRT. It occupies a key position in the network. The exclusion led to a reduction in heterogeneity and a change in the network structure, suggesting that the current evidence in the VAS aspect is relatively sensitive to individual studies and has limited robustness. As the included studies had different treatment courses, Meta-regression was performed to discuss the influence of treatment course on the efficacy. The results showed that there was no significant correlation between the results of exercise therapy and the duration of treatment compared with the control group, and the results were not statistically significant (Table 3).

**Table 3:** Results of the meta-regression analysis of the effect of duration of treatment on efficacy.

Outcome indicator	Intervening measure	Time [RR (95% CI)]
NDI	CEvsABP	−3.07 (−27.93, 26.06)
	CEvsBBAT	−11.47 (−30.58, 6.44)
	CEvsCEandTCM	−4.92 (−235.94, 76.79)
	CEvsESWTandCE	−5.42 (−148.51, 20.71)
	CEvsJOandCE	−6.18 (−29.69, 12.59)
	CEvsMET	−5.52 (−23.81, 8.75)
	CEvsNTT	−4.72 (−44.71, 15.50)
	CEvsOMTandCE	0.23 (−29.17, 32.20)
	CEvsSCEE	−6.82 (−114.89, 143.46)
	CEvsSEandCE	−3.70 (−26.52,14.49)
	CEvsTT	−0.69 (−25.98, 19.34)
	METvsMRT	2.66 (−28.66, 33.56)
VAS	CEvsCEandTCM	−1.45 (−12.25, 6.07)
	CEvsESWTandCE	−0.32 (−6.21, 5.55)
	CEvsIE	−1.04 (−5.84, 3.64)
	CEvsMET	−2.90 (−9.43, 5.14)
	CEvsSCEE	−1.05 (−24.93, 11.22)
	CEvsSEandCE	−1.13 (−5.29, 3.29)
	METvsMRT	0.77 (−7.51, 9.69)

CE, Conventional exercise; IE, isometric exercise; TT, tailored therapy; NTT, Non-tailored therapy; TE, Therapeutic exercise; MET, Muscle energy technique; MRT, Myofascial release therapy; TCM, Traditional Chinese medicine; SE, Suspension exercise; BBAT, Basic Body Awareness Therapy; OMT, Osteopathic manipulative therapy; BS, Back school; DT, Dynamic taping; ESWT, Extracorporeal shock wave therapy; JO, Jaw opening; ABP, Active behavioural physiotherapy; SCEE, Specifc cervical extensors exercise.

#### 3.4.5 Evaluation of evidence quality

The CINeMA online tool was used to evaluate the quality of evidence for the primary outcome indicators (NDI and VAS). The evidence grade was determined comprehensively from six domains: internal bias, reporting bias, indirectness, imprecision, heterogeneity, and inconsistency. In the sensitivity analysis stage, due to the insufficient number of studies meeting the “low bias risk” criteria (less than 3 studies), the model could not converge (the Fisher scoring algorithm did not converge), and thus, subgroup analysis including only studies with low bias risk could not be conducted.

## 4 Discussion

The aim of this study was to evaluate the efficacy of different exercise therapies in treating chronic nonspecific neck pain. Eventually, 17 studies were included, involving 1,224 patients and 16 intervention measures. The results showed that there was no significant difference among various exercise therapies in terms of improving function; in terms of relieving pain, compared with conventional exercise therapies, muscle energy technique (MET) showed certain advantages, but this result was mainly based on indirect comparison and the evidence network was sparse, so caution should be exercised when interpreting. In the SUCRA ranking, MET ranked high in the VAS aspect (SUCRA = 0.88), but some other intervention measures (such as BBAT, SCEE, etc.) were only supported by single small sample studies, and the stability of the ranking results was limited. It should be pointed out that although MET showed certain advantages in relieving pain, its evidence base is still insufficient. The existing clinical studies supporting MET are mostly small sample, short-term follow-up exploratory trials, and in this network Meta-analysis, most comparisons of MET with other intervention measures rely on indirect evidence, and there are limited direct comparison studies. Therefore, the results of this study should be regarded as exploratory findings rather than definitive conclusions. In clinical practice, the application of MET should be comprehensively considered in combination with the patient’s individual situation, the therapist’s experience, and contraindications, and it should not be promoted as a universal “best” intervention. The value of this study lies in systematically reviewing the evidence gaps of existing exercise therapies and providing directions for future research: it is necessary to conduct high-quality, large-sample, long-term follow-up head-to-head randomized controlled trials to verify the relative efficacy of different exercise therapies and establish an evidence-based clinical decision-making path. We conducted a Meta-regression analysis of the impact of treatment duration on efficacy, and the results showed that the impact of treatment duration on efficacy was not significant. In summary, the effect of Muscle energy technique is the best, and it can be recommended for clinical use.

MET is a manual therapy technique that uses the energy of the muscle itself to intervene. It is an active treatment technique on the premise of painless and requires the active participation of the patient. The therapist controls the direction of the lesion accurately and applies appropriate force, and the patient performs isometric or isotonic contraction of the muscle to resist the force exerted by the therapist. By relaxing and stretching muscles, the musculoskeletal system can be improved and pain can be relieved. As a manual treatment based on neurophysiological principles, the core mechanism of MET for the treatment of neck pain lies in the regulation of abnormal muscle and tendon spindle activities through precisely controlled isometric contraction-relaxation cycles. When patients actively engage in isometric contraction against the resistance imposed by therapists, the inhibitory input of Golgi tendon organs increases, while the sensitivity of muscle spindles decreases. This dual regulatory effect can effectively break the vicious cycle of pain, spasticity and pain. When MET was applied to the target muscle of CNSNP patients, the target muscle (active muscle) would produce isometric contraction to inhibit the Golgi tendon reflex, leading to reflexive relaxation of the antagonist muscle. At the same time, it also activates mechanoreceptors on the muscles around the cervical spine, stimulates the sympathetic nervous system through somatic afferents and activates the gray matter around the midbrain aqueduct to regulate the descending of pain, thereby reducing pain (38). During MET treatment, rhythmic muscle contractions of target muscle groups affect lymphatic and blood flow rates, change intercellular pressure, improve blood flow through capillaries, reduce proinflammatory cytokines, desensitise peripheral nociceptor, and reduce pain (39). MET treatment can reduce sympathetic nerve tone through fascial stimulation and relaxation of local blood vessels, reduce local edema through rhythmic muscle movement, and effectively relieve muscle spasticity and pain (40).

A number of clinical studies have confirmed that MET has a good effect on relieving neck pain. For example, Jeong et al. (41) compared the effects of passive stretching, massage, and MET on neck range of motion, muscle strength, and tenderness threshold in young people, and the results showed that MET was significantly better than the other two groups in improving neck muscle strength. Although massage had limited improvement in strength, it could significantly increase tenderness threshold, while passive stretching was better in improving cervical range of motion. In the study of Kashyap (42), manual pressure release technique (MPR) was compared with MET. All subjects received daily postural correction and neck exercise, and were divided into MPR group, MET group and routine intervention group. The results showed that MET had a significant effect on increasing tenderness threshold, and its mechanism may be related to enhancing muscle extensibility, reducing excessive muscle tension and reducing myofascial trigger point sensitivity, thus effectively relieving pain. Sadria et al. (43) compared the intervention effect of the active release technique (ART) and MET on the potential trigger points of the upper trapezius muscle. According to the evaluation of visual analogue scale (VAS), cervical lateral flexion range of motion and muscle thickness, the two techniques can both improve the relevant clinical symptoms, but there is no significant difference in the efficacy. Phadke et al. (44), for patients with mechanical neck pain, randomly divided 110 participants into the MET group and the static stretching (SS) group for a 6-day treatment. It was found that both groups could relieve pain and improve functional disability, but the MET group had more significant improvement in VAS and Neck Disability index (NDI). Buttagat et al. (45) compared the efficacy of Thai massage and MET in patients with chronic neck pain in a two-week study with a total of 8 treatments, and the evaluation indexes included pain intensity (PI), pressure pain threshold (PPT), NDI, and neck flexion range of motion (NFROM). The results showed that there was no statistically significant difference in the efficacy parameters between the two methods, indicating that both methods can be used as a treatment option for chronic neck pain. Jalal et al. (46) conducted MET training on 20 patients with neck pain accompanied by limited movement and muscle spasm for 6 months. Through the comparison of cervical range of motion and VAS score before and after treatment, it was confirmed that MET could effectively improve the range of motion of the cervical spine in all directions and reduce pain. Osama et al. (47) pointed out that in the treatment of mechanical neck pain, MET combined with joint mobilization can synergistically improve the Angle of cervical lordosis, pain, range of motion and muscle strength. This study emphasizes that, different from traditional stretching, MET targets both the active and passive components of muscles, which can not only inhibit abnormal contraction, but also achieve effective stretching. Therefore, MET has unique advantages in the treatment of chronic neck pain and upper trapezius trigger points. Subsequently, the team (48) further explored the short-term effects of static stretch (SS) and the two modes of self-inhibition (AI) and reciprocal inhibition (RI) in MET on isometric muscle strength in patients with neck pain. A total of 78 patients were divided into SS group, AI-MET group and RI-MET group. All patients received basic physical therapy, and then corresponding interventions were implemented for neck-related muscle groups. According to the evaluation of Numerical Rating Scale (NPRS) and modified Dynamometry (MSD), AI-MET was superior to SS and RI-MET in improving isometric muscle strength. In clinical practice, the implementation of MET must strictly adhere to the principle of painlessness. The therapist should precisely control the direction and intensity of the resistance to avoid triggering or exacerbating the patient’s pain. The Meta-regression results of this study show that the duration of the treatment has no significant impact on the therapeutic effect (*p* > 0.05), suggesting that the clinical benefits of MET may be more dependent on the quality of treatment, the accuracy of operation, and the patient’s compliance, rather than simply prolonging the treatment period. Therefore, it is recommended to adopt an individualized treatment strategy. Generally, a 4–6 week basic treatment course is adopted, with 2–3 sessions per week. The treatment frequency and intensity should be dynamically adjusted according to the patient’s degree of pain relief, functional improvement, and tolerance. For patients with acute severe pain, severe cervical instability, or intolerance to isometric contraction, MET should be postponed or used with caution, and inflammation and pain should be controlled first before gradually introducing it. In summary, the existing clinical evidence supports the effectiveness of MET in improving neck pain, improving cervical function and related physiological indicators, and it often shows certain advantages or non-inferiority in different comparative studies. It can be used as one of the treatment methods for neck pain rehabilitation. Currently, several clinical trial registration platforms have clinical studies of MET in the treatment of pain (e.g., ChiCTR2400087904: The effect of muscle energy technology combined with neck stability training on chronic nonspecific neck pain in college students; ChiCTR2500097441: clinical efficacy of fascial release technique combined with muscle energy technique in the treatment of postpartum low back pain; ChiCTR2200067251: Effect of muscle energy technique combined with core stability training on neuromuscular function in patients with nonspecific low back pain. NCT05040477: Muscle Energy Technique and Facet Joint Mobilization in Chronic Neck Pain; NCT04716348: Muscle Energy Technique on Forward Head Posture and Cervical Mobility in Visually Impaired Children), It indicates that MET has been recognized and concerned by more and more clinical researchers in the treatment of pain.

As a first-line intervention for non-specific neck pain, exercise therapy has a good effect. The types of exercise therapy included in Wen Yanfei’s meta-analysis (49) included muscle strength training, stability training, proprioceptive training, yoga and Pilates, etc. The control group included blank control, physical factor therapy, and health education. The results showed that exercise therapy could significantly increase the cranio-vertebral Angle (SMD = 0.84, 95%CI 0.42 to 1.26, *p* < 0.001) and reduce the visual analogue scale score (SMD = −2.05, 95%CI −2.58 to −1.52, *p* < 0.001). Increased pressure pain threshold (MD = 112.27, 95%CI 75.03–149.50, *p* < 0.001), Cervical flexion (SMD = 1.24, 95%CI 0.34–2.15, *p* = 0.007) and lateral flexion (SMD = 1.52, 95%CI 0.40–2.65, *p* = 0.008) were improved. It also improved the endurance of cervical deep flexor muscle (SMD = 1.02, 95%CI 0.10–1.94, *p* = 0.03) and the position sense of cervical joint (SMD = −1.00, 95%CI −1.470.53, *p* < 0.001). However, there was no significant effect on ROM of flexion (SMD = 0.85, 95%CI −1.04-2.75, *p* = 0.38) and rotation (SMD = 1.65, 95%CI −0.35–3.65, *p* = 0.11). Exercise therapy could also reduce the score of cervical disability index (MD = −11.88, 95%CI −16.09– −7.68, *p* < 0.001), which confirmed that exercise therapy had a significant advantage in the treatment of CNSNP. In the future, its development prospects are mainly reflected in three dimensions: (1) multimodal integration: Combined application of patient education and cognitive behavioral intervention can increase the effective rate of treatment to 75%–85% (9). Resistance training can significantly improve the pain degree of patients with chronic neck pain (VAS score reduced by 40%–60%) and the range of motion of cervical spine (ROM increased by 15–25°) (49). (2) Technological innovation: virtual reality technology can improve patient compliance by 35%–50% (9), and biofeedback system can achieve dynamic adjustment of training intensity (50). (3) The remote monitoring platform based on surface electromyography and inertial sensors provides support for home rehabilitation, and it is necessary to establish a grading and classification treatment system to solve the problem of insufficient standardization.

This study has the following methodological advantages: First, it is the first study to systematically compare the efficacy of multiple exercise therapies on CNSNP using network meta-analysis (NMA) method. NMA can provide the relative ranking of the effectiveness of different interventions (such as SUCRA value) in the absence of direct head-to-head comparison, which provides more refined evidence for clinical decision-making. Second, we strictly followed the PRISMA-NMA guidelines for reporting, and the PROSPERO platform was used to register the study protocol in advance to ensure transparency and reproducibility of the process. Thirdly, we used the Cochrane Risk of Bias Tool 2.0 (RoB 2.0) to rigorously evaluate the quality of the included randomized controlled trials, and on this basis, we conducted detailed sensitivity analysis to assess the potential impact of bias risk on the stability of the results, which enhanced the robustness of the results. Fourth, this study focused on two core outcome measures, pain intensity (VAS) and neck disability (NDI), to provide a more comprehensive view of efficacy.

The study also has several limitations. First, the included studies were heterogeneous in terms of exercise therapy protocol (e.g., intensity, frequency, duration of treatment), therapist experience, and patient baseline characteristics (e.g., pain duration, functional level), which may have obscured the true effects of specific interventions. Although we made every effort to explore sources of heterogeneity with the use of random-effects models and subgroup analyses, residual clinical and methodological heterogeneity is an important factor that needs to be treated with caution when interpreting our results. Second, most included studies lacked adequate blinding of participants and therapists, which, although difficult to avoid completely in trials of exercise interventions, can introduce performance and measurement biases, particularly with respect to patient-reported pain and functional outcomes. Third, the network evidence structure showed that direct comparison studies between some interventions (such as virtual reality training) were still scarce and the network connections were sparse. In addition, there are inherent methodological challenges in randomized controlled trials related to exercise therapy. Due to the specificity of the intervention measures, most studies have difficulty implementing blinding for participants and therapists, which may introduce implementation bias and measurement bias, especially for patient-reported outcome indicators such as pain and functional impairment. Moreover, some of the included studies have small sample sizes, which may lead to insufficient statistical power and unstable effect estimates. Although we conducted a systematic assessment using the RoB 2.0 tool, the inherent methodological limitations may still have some impact on the strength of the evidence. We verified the robustness of the core results through sensitivity analysis (by excluding studies that did not include exercise therapy). Leave-one-out analysis showed that after excluding the study by Zainab Khalid Khan, the overall comparison of each intervention measure in terms of VAS improvement changed from non-statistically significant to statistically significant (*p* < 0.05). The characteristics of this study are: a large sample size (*n* = 60), a significantly higher effect size than other similar studies (VAS change in the MET group −5.9, MRT group −5.0), and it is the only direct comparison source between MET and MR. It occupies a key connection node in the network. Its exclusion led to a change in the network structure and a reduction in heterogeneity, allowing previously concealed differences between groups to be revealed. This result indicates that the current evidence network on VAS is highly dependent on individual studies, with limited evidence robustness, and relevant conclusions should be interpreted with caution. Due to the limited number of studies included in some intervention category categories, subgroup analysis was not conducted to further control heterogeneity. In this study, some intervention measures were represented by a single small sample study but participated in the SUCRA efficacy ranking, which may result in unstable ranking results and even misleading interpretations. For example, interventions such as BBAT, SCEE, JO + CE, although have higher SUCRA values in NDI, have weak evidence bases and limited ranking credibility. Therefore, when interpreting the SUCRA results, we considered the evidence network structure and the number of included studies for stratified consideration, distinguished between “evidence sufficient” and “evidence limited” intervention measures, and avoided over-reliance on unstable ranking results. In the CINeMA evidence quality evaluation, due to the insufficient number of studies meeting the “low bias risk” criteria, a sensitivity analysis including only low bias risk studies could not be conducted, which may affect the precise assessment of the impact of bias risk. Therefore, we conducted a qualitative discussion on the impact of bias risk on the results and suggested that future studies should improve methodological quality while increasing the number of high-quality studies to support more robust evidence quality evaluation. Finally, most of the included studies were conducted in high-income countries, where the healthcare system, cultural context, and patient acceptance of exercise therapy may affect the generalizability of the results. Therefore, generalizability to other healthcare Settings should be carefully considered in light of local conditions. Future research should focus on conducting high-quality, large-sample, long-term follow-up head-to-head randomized controlled trials, and exploring the precise exercise prescription based on individual characteristics. In the future, it is necessary to strictly follow the PRISMA-NMA reporting standards, carry out RCTS with large samples and long-term follow-up, introduce individual patient data meta-analysis (IPD-NMA), and establish a dynamically updated “living” network meta-analysis system.

## 5 Conclusion

This study conducted a network meta-analysis to systematically compare the efficacy of various exercise therapies in treating chronic nonspecific neck pain. In terms of pain relief (VAS), the muscle energy technique (MET) was superior to conventional exercise therapy, and this result remained relatively stable in the sensitivity analysis, with sufficient evidence supporting it. In terms of improving neck function (NDI), there was no significant difference among all intervention measures overall. Some intervention measures had higher SUCRA values, but they were only supported by single small-sample studies, and the evidence base was weak. The conclusion should be interpreted with caution. It should be noted that in this study, some intervention measures had sparse network connections and most comparisons relied on indirect evidence. The SUCRA ranking results should be regarded as exploratory findings and are not sufficient to form clear clinical recommendations. The value of this study lies in systematically reviewing the evidence gaps of existing exercise therapies and providing directions for future research. In the future, high-quality, large-sample, and long-term follow-up head-to-head randomized controlled trials should be conducted to further verify the differences in efficacy among different exercise therapies and provide more reliable evidence support for clinical decision-making.

## Data Availability

The original contributions presented in the study are included in the article/Supplementary material, further inquiries can be directed to the corresponding authors.
