# Supplementary material for: Comparison of clinical efficacy of different exercise therapies in the treatment of chronic nonspecific neck pain: a network meta-analysis
Source: Front Neurol. 2026 Apr 13;17:1781903. doi: 10.3389/fneur.2026.1781903 (PMC13112673; doi:10.3389/fneur.2026.1781903)
Supplement: Supplementary file 2 [file Table_2.docx]

**1.Search strategy**

PubMed,Cochrane Library,Embase,CNKI,Wanfang,CBM,VIP

| **Search number** | **Query** |
| --- | --- |
| #1 | nonspecific neck pain OR non-specific neck pain[Title/Abstract] |
| #2 | exercise therapy OR exercise OR resistance training OR muscle stretching exercises [MeSH Terms] |
| #3 | core stability exercise OR motor control exercise OR virtual reality therapy OR combined exercise OR exercise therapy OR exercise OR resistance training OR muscle stretching exercises OR yoga OR core stability exercise OR motor control exercise OR virtual reality therapy OR combined exercise[Title/ Abstract] |
| #4 | #2 OR #3 |
| #5 | Randomised OR Randomized OR Blind OR phase OR multicentre OR control[All Fields] |
| #6 | #1 AND #4 AND #5 |

Web of science

| **Search number** | **Query** |
| --- | --- |
| #1 | nonspecific neck pain OR non-specific neck pain[Topic] |
| #2 | core stability exercise OR motor control exercise OR virtual reality therapy OR combined exercise OR exercise therapy OR exercise OR resistance training OR muscle stretching exercises OR yoga OR core stability exercise OR motor control exercise OR virtual reality therapy OR combined exercise[Topic] |
| #3 | Randomised OR Randomized OR Blind OR phase OR multicentre OR control[All Fields] |
| #4 | #1 AND #2 AND #3 |

**2.League table**

NDI

| **BBAT** | **SCEE** | **JOandCE** | **MET** | **ESWTandCE** | **NTT** | **SEandCE** | **MRT** | **ABP** | **CEandTCM** | **OMTandCE** | **TT** | **CE** |
| --- | --- | --- | --- | --- | --- | --- | --- | --- | --- | --- | --- | --- |
| **BBAT** | . | . | . | . | . | . | . | . | . | . | . | -11.41 (-25.41, 2.59) |
| -4.85 (-25.38, 15.68) | **SCEE** | . | . | . | . | . | . | . | . | . | . | -6.56 (-21.58, 8.46) |
| -5.49 (-24.76, 13.78) | -0.64 (-20.66, 19.38) | **JOandCE** | . | . | . | . | . | . | . | . | . | -5.92 (-19.16, 7.32) |
| -6.73 (-23.55, 10.08) | -1.88 (-19.56, 15.80) | -1.24 (-17.43, 14.94) | **MET** | . | . | . | -1.20 (-14.51, 12.11) | . | . | . | . | -4.68 (-14.00, 4.64) |
| -6.55 (-25.90, 12.80) | -1.70 (-21.80, 18.40) | -1.06 (-19.86, 17.74) | 0.18 (-16.10, 16.47) | **ESWTandCE** | . | . | . | . | . | . | . | -4.86 (-18.22, 8.50) |
| -7.01 (-26.67, 12.65) | -2.16 (-22.56, 18.24) | -1.52 (-20.64, 17.60) | -0.28 (-16.93, 16.38) | -0.46 (-19.67, 18.75) | **NTT** | . | . | . | . | . | -3.95 (-17.76, 9.86) | -4.40 (-18.21, 9.41) |
| -7.81 (-27.23, 11.61) | -2.96 (-23.13, 17.21) | -2.32 (-21.20, 16.56) | -1.08 (-17.45, 15.30) | -1.26 (-20.22, 17.70) | -0.80 (-20.08, 18.48) | **SEandCE** | . | . | . | . | . | -3.60 (-17.06, 9.86) |
| -7.93 (-29.38, 13.51) | -3.08 (-25.21, 19.04) | -2.44 (-23.40, 18.51) | -1.20 (-14.51, 12.11) | -1.38 (-22.41, 19.65) | -0.92 (-22.24, 20.40) | -0.12 (-21.22, 20.98) | **MRT** | . | . | . | . | . |
| -8.41 (-27.87, 11.05) | -3.56 (-23.77, 16.65) | -2.92 (-21.84, 16.00) | -1.68 (-18.10, 14.74) | -1.86 (-20.87, 17.15) | -1.40 (-20.72, 17.92) | -0.60 (-19.68, 18.48) | -0.48 (-21.61, 20.66) | **ABP** | . | . | . | -3.00 (-16.52, 10.52) |
| -8.76 (-27.98, 10.46) | -3.91 (-23.89, 16.07) | -3.27 (-21.94, 15.40) | -2.03 (-18.16, 14.10) | -2.21 (-20.96, 16.54) | -1.75 (-20.83, 17.33) | -0.95 (-19.78, 17.88) | -0.83 (-21.74, 20.08) | -0.35 (-19.22, 18.52) | **CEandTCM** | . | . | -2.65 (-15.81, 10.51) |
| -11.51 (-39.68, 16.66) | -6.66 (-35.35, 22.03) | -6.02 (-33.81, 21.77) | -4.78 (-30.93, 21.38) | -4.96 (-32.81, 22.89) | -4.50 (-32.57, 23.57) | -3.70 (-31.60, 24.20) | -3.58 (-32.92, 25.77) | -3.10 (-31.03, 24.83) | -2.75 (-30.51, 25.01) | **OMTandCE** | . | 0.10 (-24.34, 24.54) |
| -10.96 (-30.62, 8.70) | -6.11 (-26.51, 14.29) | -5.47 (-24.59, 13.65) | -4.23 (-20.88, 12.43) | -4.41 (-23.62, 14.80) | -3.95 (-17.76, 9.86) | -3.15 (-22.43, 16.13) | -3.03 (-24.34, 18.29) | -2.55 (-21.87, 16.77) | -2.20 (-21.27, 16.87) | 0.55 (-27.52, 28.62) | **TT** | -0.45 (-14.25, 13.35) |
| -11.41 (-25.41, 2.59) | -6.56 (-21.58, 8.46) | -5.92 (-19.16, 7.32) | -4.68 (-14.00, 4.64) | -4.86 (-18.22, 8.50) | -4.40 (-18.21, 9.41) | -3.60 (-17.06, 9.86) | -3.48 (-19.72, 12.77) | -3.00 (-16.52, 10.52) | -2.65 (-15.81, 10.51) | 0.10 (-24.34, 24.54) | -0.45 (-14.25, 13.35) | **CE** |

VAS

| **MET** | **MRT** | **CEandTCM** | **SEandCE** | **IE** | **SCEE** | **ESWTandCE** | **CE** |
| --- | --- | --- | --- | --- | --- | --- | --- |
| **MET** | -0.90 (-3.18, 1.38) | . | . | . | . | . | -2.96 (-5.39, -0.53) |
| -0.90 (-3.18, 1.38) | **MRT** | . | . | . | . | . | . |
| -1.54 (-4.90, 1.82) | -0.64 (-4.70, 3.42) | **CEandTCM** | . | . | . | . | -1.42 (-3.74, 0.90) |
| -1.81 (-5.17, 1.55) | -0.91 (-4.97, 3.15) | -0.27 (-3.56, 3.02) | **SEandCE** | . | . | . | -1.15 (-3.48, 1.18) |
| -1.93 (-5.34, 1.48) | -1.03 (-5.13, 3.07) | -0.39 (-3.72, 2.94) | -0.12 (-3.46, 3.22) | **IE** | . | . | -1.03 (-3.42, 1.36) |
| -2.53 (-5.85, 0.79) | -1.63 (-5.66, 2.40) | -0.99 (-4.23, 2.25) | -0.72 (-3.97, 2.53) | -0.60 (-3.89, 2.69) | **SCEE** | . | -0.43 (-2.70, 1.84) |
| -2.67 (-5.58, 0.24) | -1.77 (-5.47, 1.92) | -1.13 (-3.96, 1.69) | -0.86 (-3.69, 1.97) | -0.74 (-3.62, 2.14) | -0.14 (-2.92, 2.64) | **ESWTandCE** | -0.29 (-1.89, 1.32) |
| -2.96 (-5.39, -0.53) | -2.06 (-5.39, 1.27) | -1.42 (-3.74, 0.90) | -1.15 (-3.48, 1.18) | -1.03 (-3.42, 1.36) | -0.43 (-2.70, 1.84) | -0.29 (-1.89, 1.32) | **CE** |

**3.Sensitivity analysis**

NDI

VAS
